# Training Preferences of School Food Service Staff Vary by Role in the School Nutrition Program

**DOI:** 10.3390/ijerph18010050

**Published:** 2020-12-23

**Authors:** Leia Flure, Melissa Pflugh Prescott, Whitney Ajie, Trinity Allison, Jennifer McCaffrey

**Affiliations:** 1 Office of Extension and Outreach, University of Illinois at Urbana-Champaign, Urbana, IL 61801, USA; trinitya@illinois.edu (T.A.); jmccaffr@illinois.edu (J.M.); 2 Department of Food Science and Human Nutrition, University of Illinois at Urbana-Champaign, Urbana, IL 61801, USA; mpp22@illinois.edu; 3 Washington State Department of Health, Tumwater, WA 98501, USA; whitney.ajie@doh.wa.gov

**Keywords:** National School Lunch Program, nutrition, school feeding programs, school nutrition success

## Abstract

Professional development has been identified as a critical component for school nutrition professionals (SNPs) to successfully implement school meal standards in the United States. However, training needs may vary based on different factors. This study examined (1) the topics of highest priority for SNPs; (2) preferred learning methods; (3) where and when trainings should be conducted; and (4) whether responses differ according to important factors including position type, school locale (urban vs. rural), or job experience. Participants completed surveys that included questions on demographics and preferences for learning methods and training topics (*n* = 492). Descriptive statistics characterized survey responses. Chi square tests assessed differences in learning method and training topic preferences by participant role, locale, and job experience; Cramer’s V assessed the strength of association for each chi square result. Qualitative responses to open-ended questions were analyzed using an inductive thematic analysis method. Nearly all training topic preferences were significantly different (*p* < 0.001 using Bonferroni method) when stratified by role. Significant differences were also observed for school locale and years of experience, but to a lesser degree. There was less variation in learning method preferences across staff role. Qualitative results (*n* = 93) identified three key themes related to training needs: role-specific trainings, innovative learning methods, and geographic access. The combination of quantitative and qualitative analysis indicate that professional development for SNPs should mostly be conducted in-person, be easily accessible, and include hands-on activities. Further, training should be tailored by job role and address situational barriers unique to the geographic area.

## 1. Introduction

In 2010, the United States federal government updated standards for foods served in child nutrition programs through the Healthy, Hunger Free Kids Act [1]. As a result of these changes, school nutrition professionals (SNPs) have faced significant challenges. Past surveys have indicated that a large proportion of SNPs have difficulty understanding the guidelines, which included increased portion sizes for fruits and vegetables and nutrient limits, among other changes. SNPs have also struggled with barriers to implementation, such as workforce retention and lack of equipment for scratch cooking [2]. Other concerns have included menu planning with tightened sodium limits [3,4,5], poor student acceptance of whole grain foods and vegetables [6,7,8,9,10], and lack of culinary skills for serving palatable foods that meet nutrition guidelines [3]. Correspondingly, multiple surveys have identified professional development as a key need for SNPs to successfully implement federal rules for school meals [2,3,4].

To strengthen the administration of school nutrition programs, the United States Department of Agriculture (USDA) defined minimum hiring criteria for school nutrition directors and established continuing education requirements for SNPs, known as the USDA Professional Standards [11]. In effect since 2015, the USDA Professional Standards outline annual training needs in hours, with requirements varying according to a person’s role in the school nutrition program. For example, school nutrition directors must complete at least 12 h of annual continuing education while part time staff working less than 20 h per week require four hours annually. Trainings must be aligned with specific learning topics and objectives delineated by the USDA and importantly, should be relevant to the employee’s job duties. A cashier would be expected to attend trainings on topics like customer service and identifying reimbursable meals, but not topics more suited for a cook, such as knife skills or standardized recipes.

Several studies shed light on SNPs’ training needs based on knowledge, skills, and attitudes for predetermined topics; these include food allergies and other special dietary needs [12,13,14,15,16], whole grains [6,7,17], school food environment [18], farm to school [19], food hygiene and safety practices [20], and scratch cooking [21]. The few studies asking SNPs specifically about their training needs have indicated a desire for assistance with menu planning, with attention to meeting new requirements for nutrition limits and portion sizes [22,23]. Within the studies reviewed, food service directors, managers, and upper level administrators were the primary respondents [19,22]; they were either specifically targeted or there were few responses from food service workers such as cooks, servers, and cashiers. In the latter case, supervisors reported perceived training needs for their staff [22].

Overall, the limited research regarding food service workers’ topic preferences suggests they desire training in line with their job duties (e.g., cooking skills, food safety, meal pattern requirements) [23]. However, there are other factors to take into account when planning training topics for SNPs. Geographic area should be considered as it can affect resource availability [24,25]. In rural areas, for example, access to certain products is often limited and food costs may be higher as they order in smaller volumes [5,17,25,26,27]. Thus, a focus on procurement strategies could be helpful to address these barriers.

Learning methods, too, should be considered based on these factors. Face-to-face team building and skills workshops may be particularly helpful for frontline staff—especially those who work part-time and may be less experienced—to build confidence, enhance motivation, and increase the likelihood of putting knowledge and skills into practice [21,28,29]. Other learning methods such as online courses may be preferable for some learners, but to our knowledge, there is no research investigating this question specific to SNPs. Online training may also not work well for SNPs in rural areas where internet infrastructure issues cause poor connectivity [5,17,24,25,26,27].

Regardless of factors like position or location, attendance barriers such as timing and limited funds for travel and additional pay are perennial concerns [13,18,29]. Further, the lack of time for training may be worsened by labor shortages and the burdens of meal planning, preparation, and documentation [2]. It is imperative to identify the most opportune logistics to maximize attendance while minimizing strain on school nutrition programs.

Increasing participation in professional development tailored according to important factors may improve SNPs’ ability to provide school meals that meet federal standards and positively impact student nutrition. The aims of this study were to explore (1) what topics are of highest priority for school nutrition professionals; (2) what learning methods are most preferred; (3) where and when trainings should be conducted; and (4) whether responses differ according to position type, school locale, or job experience. These results will help inform planning and development of current and future training programs for SNPs. 

## 2. Materials and Methods

In 2015, the Illinois State Board of Education (ISBE) contracted with University of Illinois Extension and Outreach (Extension; part of the national cooperative extension service) to supplement their support of school nutrition programs. This partnership, named the ABCs of School Nutrition program, built infrastructure to provide ongoing statewide training opportunities. As part of the formative evaluation, Extension and ISBE conducted a survey to gather information on the training and learning preferences of Illinois school staff with child nutrition related job duties. At a later date, details about the survey and data collection were submitted to the University of Illinois Office for the Protection of Research Subjects. The study was granted a Not Human Subjects Research Determination, allowing the research team to analyze data and disseminate results without additional Institutional Review Board approval.

### 2.1. Survey Design

The survey was drafted by the research team with expert review from the Illinois School Nutrition Association and ISBE’s nutrition and wellness staff. Questions included items on demographics, learning method preferences, and training preferences (topics, timing, length).

#### 2.1.1. Demographics

Participants were asked to select from the following role types: Food service director (FSD); food service managerial (FSM—head cook, kitchen manager, etc.); all other food service staff [food service worker (FSW); server, cashier, cook, etc.]; school administration (superintendent, principal, business manager); other school staff (nurse, teacher, secretary, etc.).

Participants were given the option to disclose which school district(s) they work with. If they chose to do so, the research team matched the district name with ISBE data for number of students, number of schools, and free- and reduced-price lunch eligibility rates. Participants who did not wish to disclose their district name were presented with questions that asked them to estimate this information. For example, they could select 40% or lower, 41–59%, 60% or higher, or “I’m not sure” regarding the percentage of students eligible for free- and reduced-price lunch.

Each respondent that identified their school district was categorized as working in a rural or urban/suburban location based on publicly available information from the National Center for Educational Statistics website [30]. School districts with locale codes 11–13 and 21–23 in the database were categorized as urban/suburban while those with codes 31–33 and 41–43 were categorized as rural.

#### 2.1.2. Learning Method Preferences

Participants were asked about their preferences for different learning methods and activities, such as working in groups versus with a partner versus alone; role play or demonstrations, problem solving, or teaching others the information; online vs. face to face; etc. These items were drafted based on existing best practice information for adult learning [31] as well as feedback from in-person ABCs of School Nutrition training evaluations, and feedback from Extension educators who facilitated trainings. Items followed a Likert scale where 1 = dislike a great deal, 2 = dislike somewhat, 3 = neither like nor dislike, 4 = like somewhat, and 5 = like a great deal.

#### 2.1.3. Training Timing and Length

Regarding timing, respondents were asked to select their preferred training times by the month, day of the week, and time of day [early morning (7–10 am); later morning (10–12 pm); early afternoon (12–2 pm); later afternoon (2–4 pm)]. They were able to choose multiple preferred options for each of these. Participants were also asked about preference for length of a single training (less than one hour; 1–2 h; 2–4 h; 4 h or more) and could select multiple options.

#### 2.1.4. Training Topics

Training topic questions were divided into four sections to align with the USDA Professional Standards: Nutrition, Operations, Administration, and Communications & Marketing [32]. Participants rated how likely they would be to attend training on various topics in each category (very unlikely; somewhat unlikely; neither likely or unlikely; somewhat likely; very likely). Examples included the following: Portion sizes, reimbursable meal requirements (Nutrition); production records, procurement strategies (Operations); financial management, assessing and planning for equipment needs (Administration); food merchandising techniques, using social media to promote child nutrition programs (Communications & Marketing).

#### 2.1.5. Incentives

At the end of the survey, respondents were given the opportunity to be entered into a drawing to win one of three school nutrition banners valued at up to $200.

### 2.2. Participant Recruitment and Survey Distribution

#### 2.2.1. Recruitment

The research team did not aim for a specific number of participants, but promoted the survey to the audience of focus with the goal of collecting as many survey responses as possible within a predetermined time period of approximately 3 months (June, July, and August).

#### 2.2.2. Online Survey 

An online version of the survey was built through the Qualtrics web platform (Qualtrics, Provo, UT). The survey link was distributed through multiple channels, including ISBE’s monthly “The Outlook” e-newsletter (sent to approximately 1200 school nutrition program sponsors and 600 others who opt in) and the ABCs of School Nutrition monthly “On Your Tray” e-newsletter, which was sent to about 250 SNPs, organizational partners, and other subscribers. The link was also shared throughout the summer of 2018 on the ABCs of School Nutrition Facebook page. Printed cards with the survey link were given out to SNPs attending the Illinois School Nutrition Association and ISBE conferences in June and August 2018.

#### 2.2.3. Paper Survey

SNPs who attended professional development events led by Extension educators were given the opportunity to fill out paper versions of the survey on site. Data from the paper surveys were entered into Qualtrics manually and denoted as such to distinguish them from data obtained through the online version.

### 2.3. Inclusion/Exclusion Criteria

The primary target audience for the survey was school staff with job responsibilities related to child nutrition programs. The first question of the survey was “In your current role, do you have any duties or responsibilities related to child nutrition programs at your school or district?” Those who answered “no” were exited from the online version of the survey. Data were not included from any school employees without related job duties who might have completed the paper survey despite instructions to the contrary. Although the survey was intended for school staff within Illinois, it was not a requirement for participation.

### 2.4. Statistical Analyses

Data were analyzed with Stata (version 15.1, 2017, https://www.stata.com/). Descriptive statistics were used to characterize survey responses. Chi square tests were used to assess differences in learning method and training topic preferences by participant role, locale (urban vs rural), and job experience. Cramer’s V was used to assess the strength of association for each chi square result, with < 0.20 as weak, 0.20 ≤ X < 0.30 as moderate, and ≥ 0.30 as strong [33]. For each chi square analysis, the 5-point Likert scale was collapsed into three categories. The collapsed categories were (1) dislike a great deal or dislike somewhat, (2) neither like/dislike, and (3) like somewhat or like a great deal for learning method preferences, and they were (1) very unlikely or somewhat unlikely, (2) neither likely nor unlikely, and (3) somewhat likely or very likely for training topic preferences. Due to the large number of statistical tests used, the Bonferroni method was used to reduce the likelihood of type 1 error and α < 0.05/50 = 0.001 was used to infer significance.

### 2.5. Qualitative Analyses

Participant responses to the open-ended question, “In your own words, are there other important aspects of your training needs that you want to share?” were categorized by participant role and analyzed by a single researcher using an inductive thematic analysis method [34,35,36]. Themes were only included if they occurred across at least 4 of the 5 participant role categories and the number of responses for the theme constituted at least 10% of the total qualitative sample. Some participants provided responses that encompassed more than one theme.

## 3. Results

A total of 492 people participated in the survey. There was likely overlap among recruitment outlets where prospective participants would have viewed recruitment messages; therefore, a response rate was not calculated due to the possibility of duplication errors.

Some questions were not applicable to all survey participants and some participants chose not to answer some questions. Subsequently, the sample size for the training topic and learning method preferences ranged from 453 to 461, and the range for school demographic questions was 115 to 446. As shown in Table 1, FSDs (32.7%, *n* = 161) were the most common staff to participate in the survey, and almost 90% of participants completed the survey online (*n* = 446). The majority of SNPs completing the survey worked in urban areas (63.6%, *n* = 232) and had 501–2499 students in their district (50.4%, *n* = 58). Survey submission method (online vs. paper) significantly varied by staff role (χ^2^(3) = 181.9, *p* < 0.0001, Cramer’s V = 0.61). FSWs comprised 53% of the paper survey submissions.

### 3.1. Preferred Learning Methods

**Figure 1:**
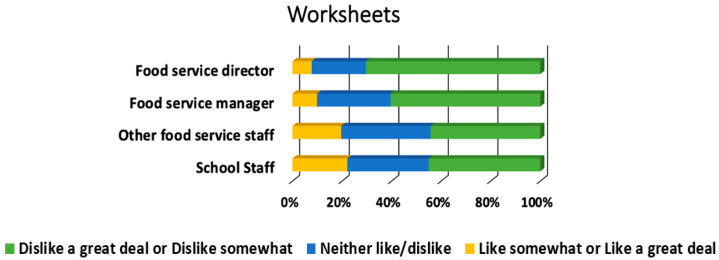
Preference for completing worksheets significantly differed by participant role. [*n* = 458 (food service director, *n* = 155; food service manager, *n* = 111; other food service staff, *n* = 61; school staff, *n* = 131), χ^2^(6) = 27.7, *p* < 0.001, Cramer’s V = 0.17].

**Figure 2:**
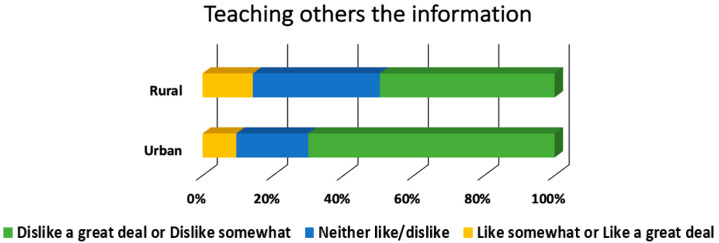
Preference for learning by teaching others significantly differed by school locale. [*n* = 361 (rural, *n* = 131; urban, *n* = 230), χ2(2) = 15.1, *p* < 0.001, Cramer’s V = 0.20].

Table 2 provides the overall results for learning method preferences. The most preferred learning methods were completing multiple activities during a training, face to face trainings, and hands-on interactive activities with 91.5% (*n* = 419), 85.7% (*n* = 394), and 85.4% (*n* = 392) respectively rating them as “like somewhat” or “like a great deal.” Conversely, role play was the least preferred learning method overall. Figure 1 shows the learning method preferences that differ by role, and Figure 2 shows the learning method preferences that differ by school locale.

### 3.2. Reported Training Topic Preferences

**Figure 3:**
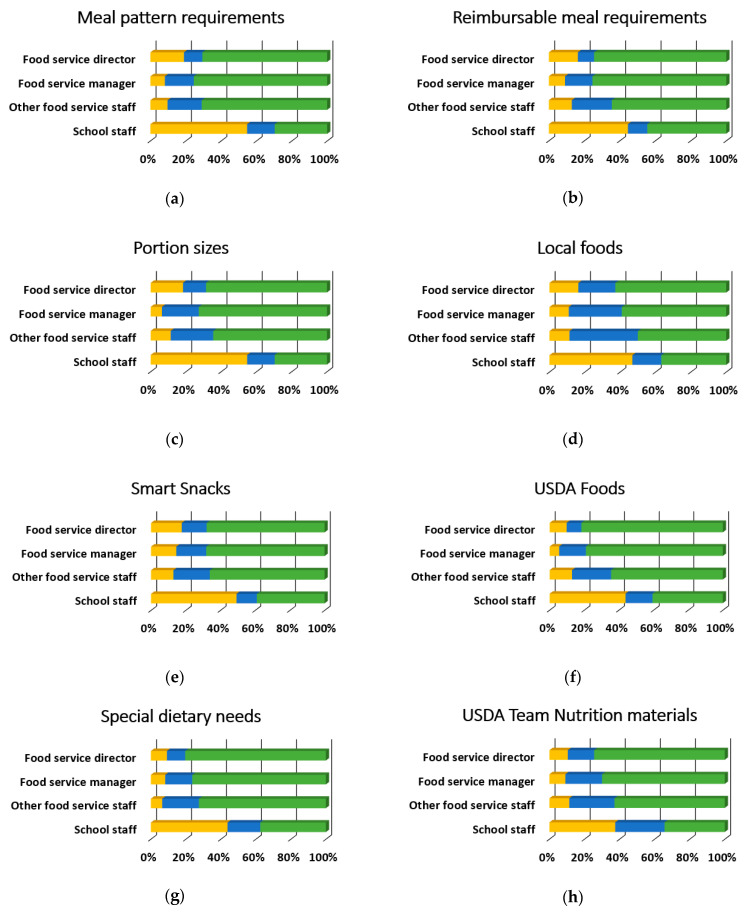

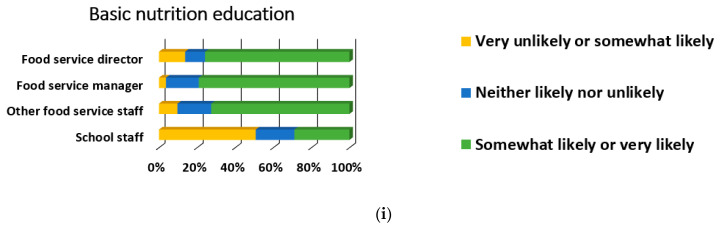
Nutrition training topic needs that significantly differed by participant role, *n* = 453 (food service director, *n* = 153; food service manager, *n* = 110; other food service staff, *n* = 62; school staff, *n* = 128). Described below for each panel are the χ^2^ and *p*-values. (**a**) Meal pattern requirements, χ^2^(6) = 96.2, *p* < 0.001, Cramer’s V = 0.33; (**b**) Reimbursable meal requirements, χ^2^(6) = 61.6, *p* < 0.001, Cramer’s V = 0.26; (**c**) Portion sizes, χ^2^(6) = 98.1, *p* < 0.001, Cramer’s V = 0.33; (**d**) Local foods, χ^2^(6) = 66.3, *p <* 0.001, Cramer’s V = 0.27; (**e**) Smart Snacks, χ^2^(6) = 56.9, *p* < 0.001, Cramer’s V = 0.25;(**f**) USDA foods, χ^2^(6) = 88.2, *p* < 0.001, Cramer’s V = 0.31; (**g**) Special dietary needs, χ^2^(6) = 90.4, *p* < 0.001, Cramer’s V = 0.32; (**h**) USDA Team Nutrition materials, χ^2^(6) = 65.7, *p* < 0.001, Cramer’s V = 0.27; (**i**) Basic nutrition education, χ^2^(6) = 114.5, *p* < 0.001, Cramer’s V = 0.36.

**Figure 4:**
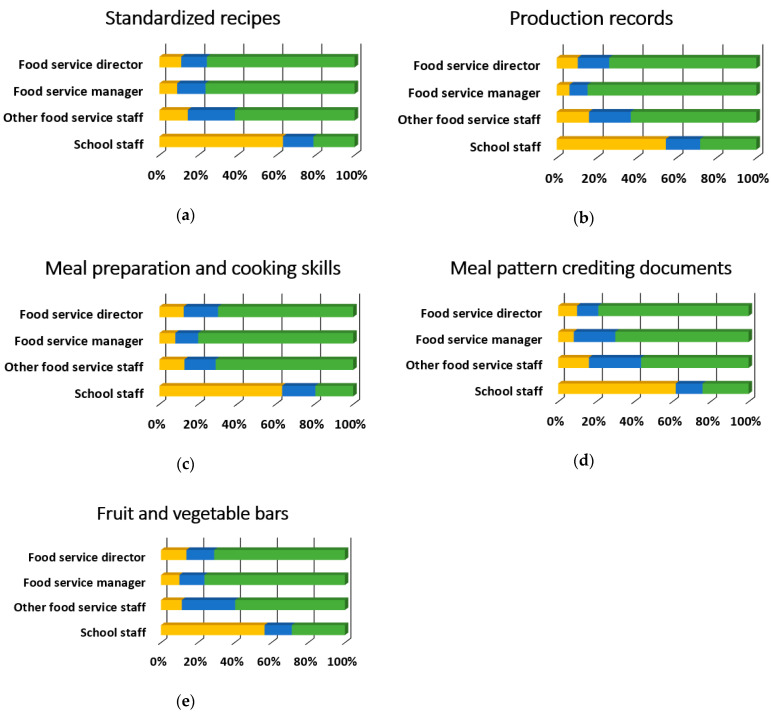

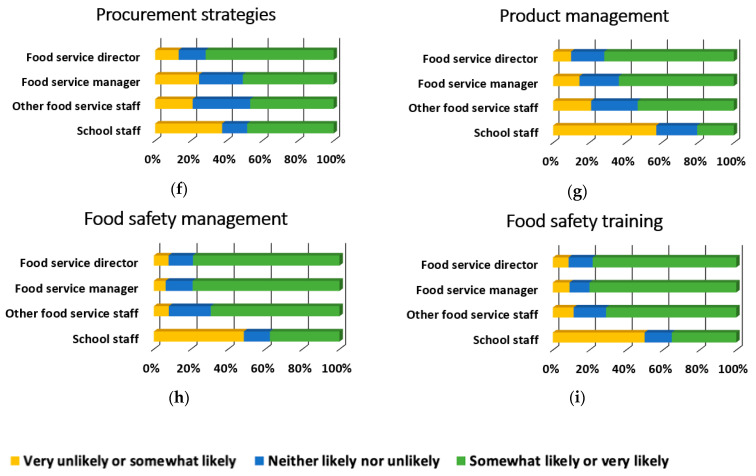
Operations training topic needs that significantly differed by participant role, *n* = 452 (food service director, *n* = 152; food service manager, *n* = 110; other food service staff, *n* = 62; school staff, *n* = 128). Described below for each panel are the χ^2^ and *p*-values. (**a**) Standardized recipes, χ^2^(6) = 144.9, *p* < 0.001, Cramer’s V = 0.40; (**b**) Production records, χ^2^(6) = 121.9, *p* < 0.001, Cramer’s V = 0.37; (**c**) Meal preparation and cooking skills, χ^2^(6) = 145.7, *p* < 0.001, Cramer’s V = 0.40; (**d**) Meal pattern crediting documents, χ^2^(6) = 145.9, *p* < 0.001, Cramer’s V = 0.40; (**e**) Fruit and vegetable bars, χ^2^(6) = 109.3, *p* < 0.001, Cramer’s V = 0.35; (**f**) Procurement strategies, χ^2^(6) = 36.9, *p* < 0.001, Cramer’s V = 0.20; (**g**) Product management, χ^2^(6) = 108.8, *p* < 0.001, Cramer’s V = 0.35; (**h**) Food safety management, χ^2^(6) = 106.7, *p* < 0.001, Cramer’s V = 0.34; (**i**) Food safety training, χ^2^(6) = 101.0, *p* < 0.001, Cramer’s V = 0.33.

**Figure 5:**
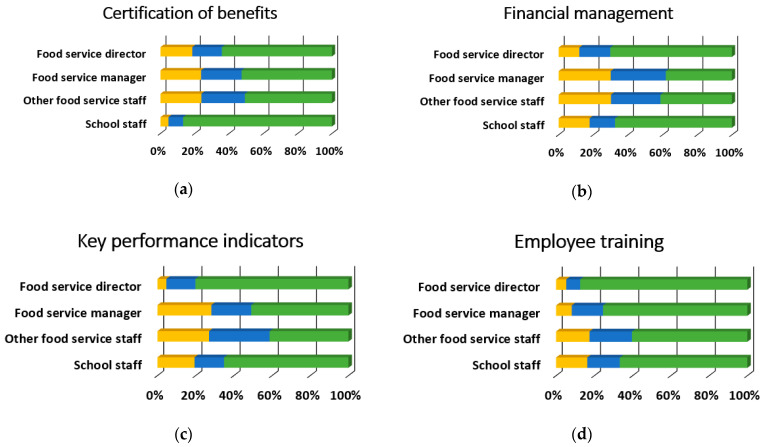

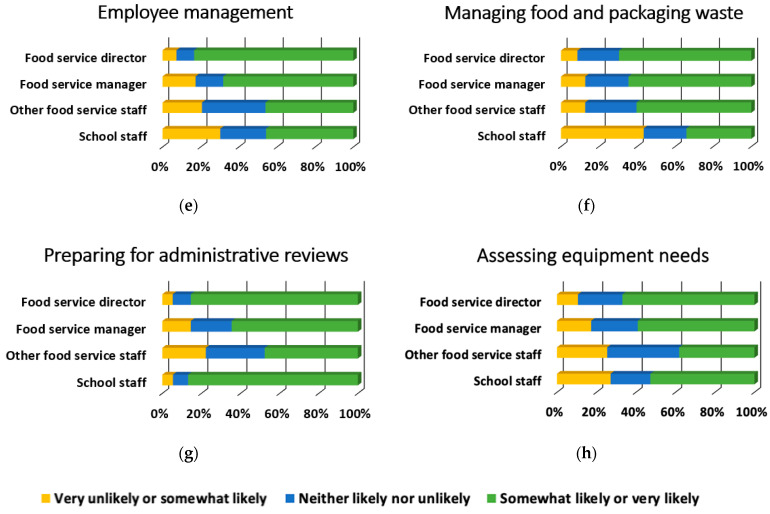
Administration training topic needs that significantly differed by participant role, *n* = 453 (food service director, *n* = 151; food service manager, *n* = 110; other food service staff, *n* = 63; school staff, *n* = 129). Described below for each panel are the χ^2^ and *p*-values. (**a**) Certification/verification of benefits (free- and reduced-price meal benefits), χ^2^(6) = 41.0, *p* < 0.001, Cramer’s V = 0.21; (**b**) Financial management (budget preparation, revenue/expenditures), χ^2^(6) = 40.4, *p* < 0.001, Cramer’s V = 0.21; (**c**) Key performance indicators to make data-driven decisions (e.g., average daily participation, meal equivalents, cost per meal equivalent, break-even point), χ^2^(6) = 46.6, *p* < 0.001, Cramer’s V = 0.23; (**d**) Employee training, procedures, and policies, χ^2^(6) = 26.2, *p* < 0.001, Cramer’s V = 0.17; (**e**) Employee management (discipline, communication, delegating, giving praise, etc.), χ^2^(6) = 57.5, *p* < 0.001, Cramer’s V = 0.25; (**f**) Managing food and packaging waste, χ^2^(6) = 67.3, *p* < 0.001, Cramer’s V = 0.27; (**g**) Preparing for administrative reviews, χ^2^(6) = 50.8, *p* < 0.001, Cramer’s V = 0.24; (**h**) Assessing and planning for equipment needs, χ^2^(6) = 23.7, *p* < 0.001, Cramer’s V = 0.16.

**Figure 6:**
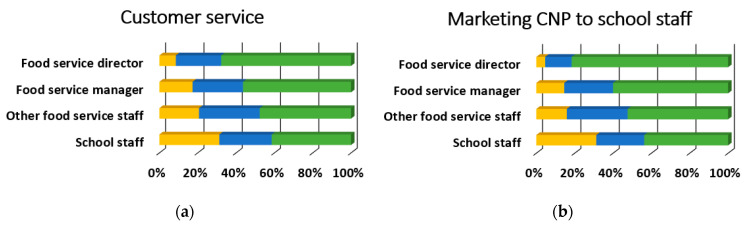

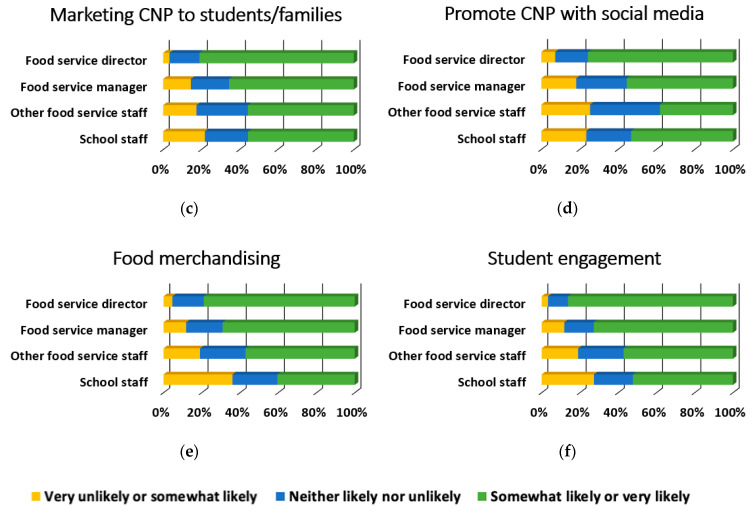
Communications and Marketing training topic needs that significantly differed by participant role, *n* = 453 (food service director, *n* = 152; food service manager, n=110; other food service staff, *n* = 63; school staff, *n* = 128). Described below for each panel are the χ^2^ and *p*-values. CNP = Child Nutrition Programs. (**a**) Customer service, χ^2^(6) = 30.0, *p* < 0.001, Cramer’s V = 0.18; (**b**) Marketing child nutrition programs to school staff, χ^2^(6) = 56.6, *p* < 0.001, Cramer’s V = 0.25; (**c**) Marketing child nutrition programs to students and families, χ^2^(6) = 31.1, *p* < 0.001, Cramer’s V = 0.19; (**d**) Using social media to promote child nutrition programs, χ^2^(6) = 34.5, *p* < 0.001, Cramer’s V = 0.20; (**e**) Smarter Lunchrooms and food merchandising techniques, χ^2^(6) = 61.7, *p* < 0.001, Cramer’s V = 0.26; (**f**) Increasing student engagement and involvement, χ^2^(6) = 49.2, *p* < 0.001, Cramer’s V = 0.23.

**Figure 7:**
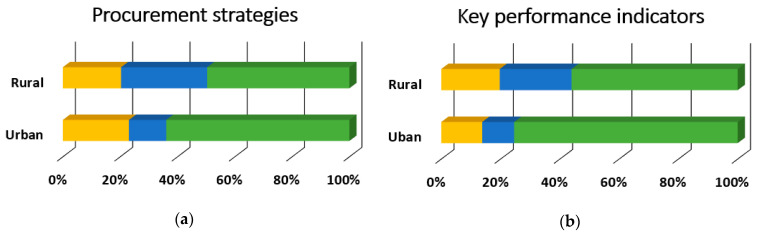

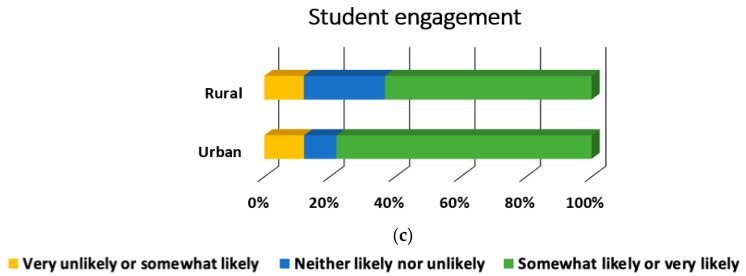
Nutrition, Operations, Administration, and Communications and Marketing training topic needs that significantly differed by school locale, *n* = 364 (rural, *n* = 133; urban, *n* = 231). Described below for each panel are the χ^2^ and *p*-values. (**a**) Procurement strategies (bidding process and writing specifications, purchasing co-operatives, etc.), χ^2^(2) = 15.9, *p* < 0.001, Cramer’s V = 0.21; (**b**) Key performance indicators to make data-driven decisions (e.g., average daily participation, meal equivalents, cost per meal equivalent, break-even point), χ^2^(2) = 16.2, *p* < 0.001, Cramer’s V = 0.21; (**c**) Increasing student engagement and involvement, χ^2^(2) = 14.6, *p* = 0.001, Cramer’s V = 0.20.

Table 3 provides the overall results for training topics. Overall, the most desired training topics were preparing for administrative reviews, employee training, procedures, and policies, and school wellness policies with 75.5% (*n* = 342), 74.83% (*n* = 339), and 72.2% (*n* = 327) respectively rating them as “somewhat likely” or “very likely.” Conversely, there were six training topics with 25% of overall respondents rating them as “somewhat unlikely” or “unlikely to attend”: meal pattern requirements, Smart Snacks, standardized recipes, meal preparation and cooking skills, meal pattern crediting documents, and product management. Nearly all of the training topics (34 out of 35) were preferred differentially by participant role (*p* < 0.001), and 85.3% (*n* = 29) of these relationships had moderate to strong effect sizes, as shown in Figure 3, Figure 4, Figure 5 and Figure 6. There was no evidence of significant differences in training topic preferences by years of job experience. Three training topics (5.6%) were preferred differentially by locale; all three had moderate effect sizes (Figure 7).

When broken down by participant role, the top five topics for FSWs were special dietary needs; basic nutrition education; meal pattern requirements; meal preparation and cooking skills; and food safety training. For FSMs, the top five topics were production records; meal preparation and cooking skills; food safety training; USDA foods; and basic nutrition education. The top five topics for FSDs were employee training, procedures, and policies; student engagement; preparing for administrative reviews; employee management; and USDA foods. The top five topics for other school staff were certification of benefits; preparing for administrative reviews; school wellness policies; financial management; and employee training.

### 3.3. Qualitative Results from Open-Ended Respones

After excluding non-substantive responses (*n* = 30), such as “none” or “I feel like you have covered it”, there were 93 requests for training needs for the open-ended question which were submitted by FSDs (*n* = 36), FSMs (*n* = 15), FSWs (*n* = 6), school administrators (*n* = 17) and other school staff (*n* = 19). As shown in Table 4, there were three themes present across four or more participant role groups. First, participants requested to have trainings that were targeted to specific roles or needs, such as those involved in cafeteria operations versus those whose job responsibilities focus on menu documentation, financial management, and other administrative duties. Similarly, participants requested trainings geared toward new staff as well as trainings that go beyond basic understanding to meet the needs of experienced staff. Second, participants requested innovation in programming to facilitate training access and convenience. Some responses that were included in this theme requested either online or in-person training, but there was no clear majority preference between the two mediums. One of the more common innovations requested was hearing from other staff or school nutrition experts, such as through a roundtable or multi-speaker webinar. Third, participants requested more locally available training options.

### 3.4. Other Training Preferences

Participants reported that June was their most preferred month to attend trainings, while December was the least desired month. The most desired days of the week for trainings were Tuesdays, Thursdays, and weekdays when there is no school (planning days, breaks, etc.). The most preferred training duration was 1–2 h, while greater than 4 h was least preferred. 

## 4. Discussion

This study examined the training needs of SNPs related to USDA Professional Standards requirements and federal rules for school meals. The results suggest an important relationship between SNPs’ role and preferences for training topics and learning methods, which was underscored by requests for role-targeted training in the qualitative analysis. School locale was also identified as a potential factor to be considered during training development. Although much of the variation in topic preferences appeared to be driven by responses from other school staff, there were clear differences among SNPs involved in the day-to-day operation of school meal programs and these aligned with typical job duties. For example, FSWs reported they were more likely to attend trainings which clustered in the Nutrition and Operations domains and included meal pattern requirements, special dietary needs, basic nutrition education, meal preparation and cooking skills, and food safety training. These matched well with topics that supervisors have reported as important for their staff [22] and topics that FSWs nationwide have most commonly selected as essential [23].

One interesting finding was that FSWs did not rate customer service highly, despite the fact that staff like servers and cashiers interact with students on a daily basis. If FSWs are less likely to seek out training specific to the topic, customer service exercises could be incorporated into other professional development opportunities they are likely to attend. Notably, a significant number of FSDs said they were not likely to attend trainings on the topics of meal pattern requirements, reimbursable meals, and portion sizes. Considering past research has documented significant concern among FSDs about implementing school meal standards [2,3,4,5,8,9,10,22,23,25,26,27] after they went into effect, it appears that some have become more comfortable with the new nutrition standards over time. This may also be related to relaxation of requirements for sodium, milk, and whole grains during that were finalized by the USDA in 2018, which has reduced pressure on FSDs to balance nutrient requirements with product availability, cost, and student acceptance [37].

There was no evidence of a significant difference in preference for online or face-to-face learning by school locale. This contrasts with previous studies reporting that poor internet access can make it difficult for rural SNPs to access online training [5,27]. At the same time, several respondents noted in the qualitative analysis that lack of internet access was an issue. Thus, it is possible that internet connectivity in rural regions has generally improved but certain areas still lag behind. Efforts should be made to offer face-to-face training regardless of location, especially because this was one of the top ranked learning methods for the general sample. Further, they should include hands-on activities, which were highly preferred regardless of role. Topics relevant to FSWs do not necessarily require an outside expert, and they may prefer to learn in the kitchen environment where they will put such information into practice.

In terms of planning and logistics, our results indicate that trainings of 1–2 h were most preferred. We did not ask SNPs about training frequency, but others have reported a preference for training up to 11 times per year [20]. Thus, it may be better received and more effective for SNPs to engage in shorter trainings, perhaps on a monthly basis, or slightly longer trainings every few months. Others have suggested that professional development be offered during the work week and ideally during the regular workday [29]. However, this may not be possible due to constraints of daily meal service and many programs lack funds to pay staff overtime for training outside of normal work hours. Thus, trainings should be delivered in flexible, innovative ways, which was a major theme in the qualitative analysis. Qualitative responses also highlighted the desire for trainings to be regionally accessible, so they should also be offered at convenient locations to minimize time and funds needed for travel.

One limitation of the current study is that age was not considered as a potential factor impacting learning preferences. SNPs from older generations have been shown to prefer face-to-face training rather than online [20], so this should be included in future studies. The study did not investigate how demographic factors could interact; for example, FSWs in urban districts may have different needs than those in rural districts, especially if they have varying levels of experience. Preferences for training topics and learning methods could be further narrowed with additional statistical analyses or by focusing recruitment on more specific audiences.

It was challenging to obtain significant representation from FSWs, who made up about 13% of respondents. This was greater than the 8% represented in Jones et al.’s study [22], but similar to the 14% response rate for FSWs in a nationwide survey conducted by the Pew Charitable Trusts and the Robert Wood Johnson Foundation [23]. Our survey was initially sent out through electronic communications; when the research team noticed that primarily supervisors and administrators were completing it, Extension educators were asked to distribute paper copies during on-site trainings. It is possible that educators did not ask SNPs to complete the survey at trainings, or they may have neglected to mail collected surveys to the research team. Also, the majority of these trainings were conducted at smaller schools with fewer SNPs. Additional responses from this subset could be elicited by having surveys available at larger trainings, such as the state board of education’s annual back-to-school nutrition conference. Surveys could also be completed directly through the Qualtrics website on laptops or tablet devices to streamline data collection. Different incentives may also be needed to motivate FSWs to complete the survey, since the marketing banners we offered as drawing prizes may have appealed more to FSMs and FSDs.

In the future, specific efforts should also be made to obtain a more geographically diverse sample. Due to limitations in recruitment, the results may not apply to all SNPs within the state. Further, our study was conducted in the Midwest and cannot necessarily be generalized to SNPs in different regions of the United States.

It should be noted that preferences were rated based on likelihood of attending training on a given topic and may better reflect training wants rather than true needs. This could be addressed in future studies by asking SNPs more specifically to list topics for which they need training or have them rate topics by importance. Further, reported training needs could be compared to administrative review reports to investigate whether perceptions of training needs match with commonly cited issues across school nutrition programs. For the current study, we did not evaluate the effectiveness of various learning methods for school nutrition training, but future research may investigate whether preferred learning methods are also more effective in terms of knowledge retention and implementation.

## 5. Conclusions

This study adds to the body of literature regarding training needs of SNPs and importantly, contributes more specific information about learning preferences depending on various factors. Based on our results, professional development for SNPs should mostly be conducted in-person, be easily accessible, include hands-on activities. Training should also be tailored by job role and address unique situational barriers. Online learning may serve as a useful adjunct for those who prefer to work independently, are comfortable with technology, and have reliable internet access.

## Figures and Tables

**Table 1:** Characteristics of the Training & Learning Preferences Survey for Illinois Child Nutrition Program Survey participants.

Characteristics	Frequency (%)
Role in child nutrition: (*n* = 492)	
Director	161 (32.7)
Managerial	117 (23.8)
All other foodservice staff	65 (13.2)
Administration	68 (13.8)
Other staff	81 (16.5)
Years of experience in child nutrition: (*n* = 491)	
<3	73 (14.9)
3–5	83 (17.0)
5–10	93 (18.9)
>10	242 (49.3)
Neighborhood area type: (*n* = 365)	
Rural	133 (36.4)
Urban	232 (63.6)
Survey method: (*n* = 492)	
Paper	86 (17.5)
Web	406 (82.5)
# of students in district: (*n* = 115)	
<500	35 (30.4)
501–2499	58 (50.4)
2500–9999	8 (7.0)
>10,000	7 (6.1)
Unsure	7 (6.1)
Offers National School Lunch Program: (*n* = 446)	
No	13 (2.9)
Yes	433 (97.1)
Offers School Breakfast Program: (*n* = 446)	
No	115 (25.8)
Yes	331 (74.2)
Offers Child and Adult Care Food Program: Early childhood meals: (*n* = 446)	
No	417 (93.5)
Yes	29 (6.5)
Offers Child and Adult Care Food Program: Supper program: (*n* = 446)	
No	437 (98.0)
Yes	9 (2.0)
Offers Summer Food Service Program: (*n* = 446)	
No	395 (88.6)
Yes	51 (11.4)
Offers Seamless Summer Option: (*n* = 446)	
No	400 (89.7)
Yes	46 (10.3)
Offers Afterschool Snack Program: (*n* = 446)	
No	374 (83.9)
Yes	72 (16.1)
Offers Special Milk Program: (*n* = 446)	
No	383 (85.9)
Yes	63 (14.1)
Offers Community Eligibility Provision: (*n* = 446)	
No	373 (83.6)
Yes	73 (16.4)

**Table 2:** Reported Preferred Learning Methods of Illinois Child Nutrition Program staff.

Learning Preferences: Like to Attend Training on/with:	Frequency (%)				
	Dislike a Great Deal	Dislike Somewhat	Neither Like/Dislike	Like Somewhat	Like a Great Deal
Interactive Activity: (*n* = 459)	9 (2.0)	10 (2.2)	48 (10.5)	146 (31.8)	246 (53.6)
Peer to Peer Sharing: (*n* = 461)	13 (2.8)	18 (3.9)	82 (17.8)	171 (37.1)	177 (38.4)
Training by Myself: (*n* = 460)	22 (4.8)	30 (6.5)	122 (26.5)	170 (37.0)	116 (25.2)
A Partner: (*n* = 459)	7 (1.5)	25 (5.5)	100 (21.8)	210 (45.7)	117 (25.5)
With a Group: (*n* = 459)	18 (3.9)	38 (8.3)	89 (19.4)	198 (43.1)	116 (25.3)
Multiple Activities during Training (*n* = 461)	7 (1.5)	17 (3.7)	84 (18.2)	188 (40.8)	165 (35.8)
Hear from an Expert: (*n* = 458)	-	4 (0.9)	35 (7.6)	125 (27.3)	294 (64.2)
Training Online: (*n* = 460)	13 (2.8)	40 (8.7)	67 (14.6)	159 (34.6)	181 (39.4)
Face to Face Learning: (*n* = 460)	4 (0.9)	5 (1.1)	57 (12.4)	160 (34.8)	234 (50.9)
Learn on Text with Many Pictures: (*n* = 459)	29 (6.3)	45 (9.8)	144 (31.4)	153 (33.3)	88 (19.2)
Watching Videos: (*n* = 461)	12 (2.6)	30 (6.5)	104 (22.6)	189 (41.0)	126 (27.3)
Learn by Physical Movement: (*n* = 459)	3 (0.7)	15 (3.3)	124 (27.0)	170 (37.0)	147 (32.0)
Learn by Teaching others the Material: (*n* = 459)	17 (3.7)	41 (8.9)	128 (27.7)	167 (36.2)	109 (23.6)
Quizzes and Knowledge Check: (*n* = 460)	17 (3.7)	54 (11.7)	135 (29.4)	171 (37.2)	83 (18.0)
Worksheets: (*n* = 458)	19 (4.2)	45 (9.8)	132 (28.8)	172 (37.6)	90 (19.7)
Learn with Problem-Solving or Brainstorming: (*n* = 460)	11 (2.4)	20 (4.4)	102 (22.2)	184 (40.0)	143 (31.1)
Role Play or Demonstration: (*n* = 460)	47 (10.2)	69 (15.0)	128 (27.8)	125 (27.2)	91 (19.8)

**Table 3:** Reported Training Topic Preferences of Illinois Child Nutrition Program staff.

Training Topics	Frequency (%)				
	Very Unlikely	Somewhat Unlikely	Neither Likely Nor Unlikely	Somewhat Likely	Very Likely
**Nutrition:**					
Portion Sizes: (*n* = 453)	71 (15.7)	41 (9.1)	78 (17.2)	142 (31.4)	121 (26.7)
Local Foods: (*n* = 453)	64 (14.1)	40 (8.8)	110 (24.3)	139 (30.7)	100 (22.1)
Smart Snacks: (*n* = 453)	75 (16.6)	39 (8.6)	69 (15.2)	157 (34.7)	113 (24.9)
USDA Foods: (*n* = 453)	59 (13.0)	26 (5.7)	64 (14.1)	172 (38.0)	132 (29.1)
Special Dietary Needs: (*n* = 453)	47 (10.4)	36 (8.0)	70 (15.5)	160 (35.3)	140 (30.9)
USDA Team Nutrition materials (*n* = 453)	46 (10.2)	35 (7.7)	98 (21.6)	158 (34.9)	116 (25.6)
Basic Nutrition Education: (*n* = 453)	63 (13.9)	33(7.3)	72(15.9)	142 (31.4)	143(31.6)
**Operations:**					
Standardized Recipes: (*n* = 452)	86 (19.0)	31 (6.9)	71 (15.7)	128 (28.3)	136 (30.1)
Production Records: (*n* = 452)	72 (15.9)	31 (6.9)	69 (15.3)	144 (31.9)	136 (30.1)
Meal Preparation and Cooking Skills: (*n* = 452)	83 (18.4)	34 (7.5)	72 (15.9)	130 (28.8)	133 (29.4)
Meal Pattern Crediting Documents: (*n* = 452)	79 (17.5)	34 (7.5)	76 (16.8)	137 (30.3)	126 (27.9)
Fruit and Vegetable Bars: (*n* = 452)	71 (15.7)	40 (8.9)	75 (16.6)	138 (30.5)	128 (28.3)
Responsibilities at Point-of-Service: (*n* = 452)	44 (9.7)	26 (5.8)	83 (18.4)	151 (33.4)	148 (32.7)
Procurement Strategies: (*n* = 452)	63 (13.9)	45 (10.0)	88 (19.5)	114 (25.2)	142 (31.4)
Product Management: (*n* = 452)	71 (15.7)	46 (10.2)	97 (21.5)	136 (30.1)	102 (22.6)
Food Safety Management: (*n* = 452)	59 (13.1)	27 (6.0)	68 (15.0)	154 (34.1)	144 (31.9)
Food Safety Training: (*n* = 452)	64 (14.2)	30 (6.6)	62 (13.7)	131 (29.0)	165 (36.5)
**Administration:**					
Certification or Verification of Benefits: (*n* = 453)	42 (9.3)	33 (7.3)	79 (17.4)	121 (26.7)	178 (39.3)
School Wellness Policies: (*n* = 453)	27 (6.0)	21 (4.6)	78 (17.2)	161 (35.5)	166 (36.6)
Financial Management: (*n* = 453)	51 (11.3)	42 (9.3)	99 (21.9)	112 (24.7)	149 (32.8)
Key Performance Indicators to make Data Driven Decisions: (*n* = 453)	42 (9.3)	38 (8.4)	86 (19.0)	128 (28.3)	159 (35.1)
Employee Training Procedures and Policies: (*n* = 453)	32 (7.1)	17 (3.8)	65 (14.4)	166 (36.6)	173 (38.2)
Training on Employee Management: (*n* = 453)	52 (11.5)	30 (6.6)	82 (18.1)	141 (31.1)	148 (32.7)
Managing Food and Packaging Waste: (*n* = 453)	62 (13.7)	29 (6.4)	104 (23.0)	150 (33.1)	108 (23.8)
Preparing for Administrative Reviews: (*n* = 453)	27 (6.0)	18 (4.0)	66 (14.6)	127 (28.0)	215 (47.5)
Assessing and Planning for Equipment Needs: (*n* = 453)	52 (11.5)	34 (7.5)	109 (24.1)	139 (30.7)	119 (26.3)
**Communication and Marketing:**					
Customer Service: (*n* = 453)	48 (10.6)	37 (8.2)	120 (26.5)	144 (31.8)	104 (23.0)
Marketing Child Nutrition Programs to School and Staff: (*n* = 453)	49 (10.8)	24 (5.3)	101 (22.3)	160 (35.3)	119 (26.3)
Marketing Child Nutrition Program to Students and Families: (*n* = 453)	40 (8.8)	20 (4.4)	92 (20.3)	169 (37.3)	132 (29.1)
Social Media to Promote Child Nutrition Programs: (*n* = 453)	46 (10.2)	31 (6.8)	108 (23.8)	150 (33.1)	118 (26.1)
Smarter Lunchrooms and Food Merchandising Techniques: (*n* = 453)	51 (11.3)	27 (6.0)	91 (20.1)	166 (36.6)	118 (26.1)
Improving Computer Skills: (*n* = 453)	60 (13.3)	32 (7.1)	113 (24.9)	130 (28.7)	118 (26.1)
Increasing Student Engagement and Involvement: (*n* = 453)	47 (10.4)	18 (4.0)	74 (16.3)	155 (34.2)	159 (35.1)

**Table 4:** Qualitative analysis findings of the open-ended survey question (*n* = 93) ^1^.

Theme	Participant Roles * Included	Representative Quotes
Desire for role-targeted or need-specific training (*n* = 55)	FSD = 24 FSM = 9 FSW = 2 SA = 8 Other school staff = 12	“I want to meet the annual training requirements in a way that’s relevant to my job. We contract with a food service management company so it doesn’t make sense for me to get in the weeds on menu planning and dietary calculations, but I would be very interested in the Operational and Communication training topics.” (SA) “New employees could use more training overall with details to review kitchen procedures, regulations and menus. “(SA) “Would like to see two different sessions: one for those who are actually involved in the cafeteria operations i.e., managing, food preparation, serving, etc. and the other for those involved in the program application, direct certification, free/reduced lunch applications, reimbursement, and verification processes. In our district, the cafeteria operations are contracted with an outside agency, but I am responsible for the program application, direct certification, free/reduced lunch applications, reimbursement, and verification processes. I have no need to attend a two-day conference when over 50% of the sessions do not impact my responsibilities.” (Other school staff)
Desire for innovation in the training mode to facilitate access and convenience (*n* = 28)	FSD = 11 FSM = 5 FSW = 4 SA = 4 Other school staff = 4	“I think each district is so unique, but in a Director’s position we are often able to leave our district with having disruption in operation. Training that would allow us to attend and provide somewhat [of] a train the trainer model that we can then, in turn, come back and train staff would be hugely beneficial from my perspective.” (FSD) “The trainings for Professional Standards hours for employees...I get that you say it is everywhere but it is not always as easy as you say … some of my staff believe it or not does not have internet access of any kind at home, and in a typical work day [they] are busy from the time they clock in till the time they clock out. [Another] issue is them getting upset of having more to do when [there] is only so many hours in a work day and the pay is already low scale… They feel like you just keep wanting more and more and it becomes a bit overwhelming for the pay they receive, and the limited time they already have. I have literally had to give trainings to mine while we were eating lunch.” (FSM)
Desire for locally available training (*n* = 12)	FSD = 2 FSM = 1 FSW = 0 SA = 5 Other school staff = 4	“Because the guidelines change, students’ acceptance of new foods change, nutritional needs change, I feel it is important for the state to offer training in various categories of nutrition. And offer it in areas throughout the state... Many rural schools (probably the ones that have the least amount of help with their nutrition issues) cannot always travel 200 miles for a 4 h training. Perhaps if there were trainers located throughout the state that could hold mini seminars in different areas (quadrants) of the state, a larger food service population could be served.” (FSD) “I think training should be done locally, not just in Springfield.” (FSD) “We love all the training that there can be but, it would be nice if some of it was held closer to where we live like Rockford or Freeport or Sterling. We cannot all travel to Springfield all the time. Or into the Chicago area. It would be nice to be noticed as a small district here in Northern Illinois/Northwest Illinois. (FSM)

^1^ Participants were asked, “In your own words, are there other important aspects of your training needs that you want to share?” * FSD = Food Service Director, FSM = Food Service Manager, FSW = Other Food Service Staff, SA = School administrators.
